# Towards Application-Driven Task Offloading in Edge Computing Based on Deep Reinforcement Learning

**DOI:** 10.3390/mi12091011

**Published:** 2021-08-26

**Authors:** Ming Sun, Tie Bao, Dan Xie, Hengyi Lv, Guoliang Si

**Affiliations:** 1 College of Computer Science and Technology, Jilin University, Changchun 130012, China; baotie@jlu.edu.cn (T.B.); xiedan@jlu.edu.cn (D.X.); 2 Changchun Institute of Optics, Fine Mechanics and Physics, Chinese Academy of Sciences, Changchun 130033, China; lv_hengyi@163.com (H.L.); siguol@163.com (G.S.)

**Keywords:** task offloading, edge computing, application-driven task, deep reinforcement learning

## Abstract

Edge computing is a new paradigm, which provides storage, computing, and network resources between the traditional cloud data center and terminal devices. In this paper, we concentrate on the application-driven task offloading problem in edge computing by considering the strong dependencies of sub-tasks for multiple users. Our objective is to joint optimize the total delay and energy generated by applications, while guaranteeing the quality of services of users. First, we formulate the problem for the application-driven tasks in edge computing by jointly considering the delays and the energy consumption. Based on that, we propose a novel Application-driven Task Offloading Strategy (ATOS) based on deep reinforcement learning by adding a preliminary sorting mechanism to realize the joint optimization. Specifically, we analyze the characteristics of application-driven tasks and propose a heuristic algorithm by introducing a new factor to determine the processing order of parallelism sub-tasks. Finally, extensive experiments validate the effectiveness and reliability of the proposed algorithm. To be specific, compared with the baseline strategies, the total cost reduction by ATOS can be up to 64.5% on average.

## 1. Introduction

With the increasing amount and variety of application data, users’ demand for high-quality services has been growing. As a new computing model of IoT, edge computing has become a highly virtualized platform to provide computing, storage, and network services between terminal devices and traditional cloud data centers. As an important infrastructure of edge computing network, edge node includes the switch, router, and embedded server. With the continuous development of Internet terminal devices, smartphones and other terminal devices are widely used. So far, the penetration rate of smartphones in the United States has reached 80%. According to the results released by Cisco, the average number of connected devices per capita will reach 3.6 by 2023 [1]. In edge computing, due to the increasing variety and quantity of data producing from IoT devices, the demand of end-users for high-quality mobile services also increases. In addition, due to the increasing number of connected devices on edge nodes, insufficient resource supply will also lead to high costs and serious load imbalance between edge nodes. Therefore, a complete and comprehensive task offloading strategy is particularly critical for the development of the edge computing network and better application performance promotion.

**Figure 1:**
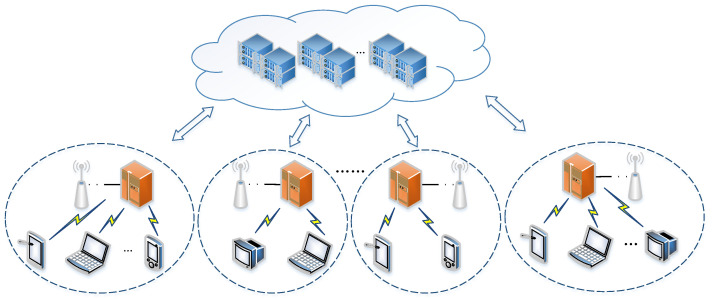
An overview of the system architecture.

Figure 1 shows the overview of the system architecture that referring to in this paper. We suppose that the system architecture contains three layers which are cloud, edge, and user. In the edge layer, there are several edge nodes with different limited capacities. We suppose that the connections and locations of the edge nodes have been fixed by the third-party service providers or the cloud data centers. For each user, the connection scope is within a certain area, as shown in the dotted circle in Figure 1. In this area, users can offload tasks to the corresponding edge node or process through a local device. This paper studies the application-driven tasks constructed by several sub-tasks with strong dependencies, and the sub-tasks that belong to one application can process on different devices. In our system architecture, the provisioning process of the application is to decide the locations of sub-tasks. For example, we use $A_{i}$ to denote the $i^{th}$ application that contains three sub-tasks: $a_{1}$, $a_{2}$, and $a_{3}$. The dependencies of these sub-tasks is $a_{1}\to a_{2}\to a_{3}$. One extreme solution is offloading strategy by minimizing the transmission cost for $A_{i}$, which processes all sub-tasks on local devices with the order $a_{1}\to a_{2}\to a_{3}$. However, this solution has the highest delay due to the limited capacity of local devices. Another extreme solution is the offloading strategy by minimizing the processing delay for $A_{i}$, which processes all sub-tasks on edge nodes. The user transmits the sub-tasks to the edge node for processing through the wireless channel, and the results are returned after all sub-tasks are completed. However, when the sizes of sub-tasks are large, the total transmission cost will be correspondingly large due to the dependencies between the precursor and the successor, which will lead to a decreasing in the quality of service of users. In this paper, we propose an efficient offloading strategy for multiple users that jointly considering the processing delay and transmission cost.

In this paper, we concentrate on the application-driven task offloading problem in edge computing by considering the strong dependencies of sub-tasks. The most important point under this problem is to determine the offloading locations of sub-tasks for users, so as to joint optimize the total delay and energy consumption generated by applications while guaranteeing the quality of services for users. Our problem poses several unique challenges as follows: (i) Since the capabilities of edge nodes and local devices are limited and different, it is nontrivial that finding a feasible strategy to complete the sub-task within improving the total cost for users during the offloading process. (ii) In our problem, we consider the applications with strong dependencies which can not achieve complete parallelism. Thus, it is challenging to find a solution that satisfies the dependency relationship with lower cost. The major novel contributions of this paper are as follows:

- We discuss the offloading problem for the application-driven tasks in edge computing, and we optimize the total cost of users which jointly consider the delays and energy consumption.
- We propose an application-driven task offloading strategy (ATOS) based on deep reinforcement learning (DRL) by adding a preliminary sorting mechanism to realize the joint optimization of the delays and energy consumption. Specifically, we analyze the characteristics of application-driven tasks and propose a heuristic algorithm by introducing a new factor to determine the processing order of parallelism sub-tasks. Based on that, we propose a task offloading strategy based on the deep Q-learning network (DQN) by training a fully connected neural network.
- We design a simulator to evaluate our strategy ATOS by comparing it with several state-of-the-art ones. The results are presented from different perspectives to provide conclusions.

The remainder of this paper is organized as follows. Section 2 surveys related works. Section 3 describes the model and then formulates the problem. Section 4 investigates the application-driven task offloading strategy based on DRL. Section 5 presents the experiments. Finally, Section 6 concludes the paper.

## 2. Related Work

The concept of edge computing was introduced to extend the cloud to the edge of the network, thus enabling a new breed of applications and services. There are numerous novel strategies on the task offloading problem in edge computing that have been proposed. Mao et al. [2,3] introduced an online learning assignment strategy based on dynamic computing offloading that is applicable to a single user. During the offloading process, the execution cost (time delay and offload failure rate) of performing the offloading was measured at each time interval. The online learning allocation strategy only depends on the current system state and did not need to calculate the task feedback results, the distribution information of the wireless channel, and the energy collection. Chen et al. [4] discussed the solution of moving edge computing to meet the low latency requirements in an ultra-dense network. Using the idea of a software-defined network, the task offloading problem was expressed as a mixed-integer nonlinear computing process, and the delay reduction problem was transformed into two sub-problems: task offloading problem and resource allocation problem. Yu et al. [5] considered the application scenarios of the IoT (Internet of Things) and reduced the computing delay by allocating resources reasonably for the program. Then, a complete polynomial-time approximation scheme was proposed, which was more effective in shortening the computing delay than the heuristic algorithm. Spatharakis et al. [6] proposed a two-level Edge Computing architecture to offer computing resources for the remote execution of location based services (LBS). Xu et al. [7] proposed a distributed computing offloading algorithm designed with the method of game theory, and the calculation delay index was quantified to achieve a lower calculation time cost.

In recent years, with the continuous development of machine learning methods, it has gradually infiltrated into various fields, among which reinforcement learning has also found a good application in the offloading decision to reduce the time delay. Meng et al. [8] proposed a delay-sensitive task offloading algorithm based on deep reinforcement learning (DRL) to improve the task processing speed and reduce the task timeout. A new reward function was designed to guide the algorithm to learn offloading decisions from the environment by combining timeout signals and deceleration signals. In addition, intelligent algorithms have also been applied to various fields. Li et al. [9] proposed a joint optimization method of task allocation ratio, channel bandwidth, and computing resources of mobile edge servers based on genetic algorithm, aiming at the situation that part of computing tasks can be partially allocated to the mobile edge server. Under the constraints of wireless transmission resources and mobile edge server processing resources, a genetic algorithm is used to solve the optimization problem of minimizing user task completion time, and the optimal offloading task strategy and resource allocation scheme were obtained. All the above offloading decisions have achieved the purpose of reducing time delay, but they failed to consider the energy consumption at the end of terminal devices during the calculation of the task offloading. The terminal devices may not be able to operate normally due to the lack of power, which has a huge impact on users’ experience.

There are also many solutions for the task offloading problems in different environmental scenarios from the perspective of optimizing energy consumption. Zhang et al. [10] adopt the artificial fish swarm algorithm to design the offloading strategy for energy consumption optimization under the constraint of delay. This strategy takes full account of the link conditions in the task data transmission network and effectively reduces the energy consumption of the equipment. However, this strategy has the defect of too high algorithm complexity. In a multi-resource environment, Xu et al. [11] designed an energy-minimization particle swarm task scheduling algorithm for multi-resource matching to reduce the energy consumption of edge terminal devices. Wei et al. [12] proposed that the task offloading problem can be divided into mobile management and energy-saving optimization, and they use a greedy algorithm to minimize the energy consumption of mobile devices. Lu et al. [13] focus on minimizing the total cost for multiple mobile users to provide an efficient resource provisioning scheme by considering three different cases in edge computing. Yu et al. [14] studied the problem of task offloading in ultra-dense network scenarios. They proposed a task offloading algorithm based on Lyapunov optimization theory, which minimizes the overall energy consumption of the base station and equipment. In order to solve the privacy leakage problem that may occur in the computing offloading decision of mobile edge computing, Zhao et al. [15] proposed a privacy perception computing offloading algorithm. This algorithm has low computational complexity and maintains low terminal energy consumption while ensuring high privacy security. Liu et al. [16] studied the offloading problem based on deep learning, and they proposed a group sparse beamforming framework to optimize network power consumption.

Some studies jointly considered the energy consumption and delay in offloading trade-off optimization problems and put forward some ideas and solutions. Zhang et al. [17] proposed an offloading mechanism assisted by SDN-V, which is suitable for the scenario of the IoV (Internet of Vehicles). The mechanism considered the task diversity, establishes the mathematical model of importance degree, and designed the task offloading sorting algorithm according to the model. Finally, an offloading algorithm based on Q-learning is constructed to optimize the energy consumption and time delay during task offloading. In the case of mobile edge computing, there are many reinforcement learning methods to solve optimization. Zhang et al. [18] proposed a policy-based DRL scheme to solve the problem that a single mobile device offloads tasks to a single mobile edge server. However, there is a question of how much to tweak the network each time that the policy is updated. Too large an amplitude may lead to the problem of non-convergence, while too small an amplitude may lead to the problem of slow convergence. Song et al. [19] proposed a semi-online computational offloading model soCoM based on dueling deep-Q network to explore the user behaviors in sophisticated action space by reinforcement learning for catching unknown environment information. Liu et al. [20] proposed an improved scheme. In this scheme, an artificial neural network was firstly used to learn strategies and make decisions, and another artificial neural network was used to score this decision [21]. In order to improve this problem, Zhan et al. [22] proposed a scheme of disengagement strategy. Firstly, two artificial neural networks were used to approximate the behavior strategy and the target strategy respectively. Then, learning data was generated from behavioral strategies to train the neural network of target strategies. Finally, the parameters of the trained target policy were assigned to the behavior policy. After repeated iterative learning of the target strategy [23], it introduced more artificial neural networks and more parameters. In this paper, we are committed to designing an offloading strategy based on DRL for application-driven tasks that jointly optimize the total delay and energy consumption.

## 3. Model and Problem Formulation

In this section, we first describe our system model which includes the application model, execution model, and transmission model. Then, we present our problem formulation.

### 3.1. System Model

The system model is abstracted by the architecture in Figure 1, which is constructed by three layers. The cloud layer is located at the top that is a core in the whole system model which is far from the users. In order to avoid the long-distance transmission and relieve the pressure of the cloud, this paper considers the offloading decision of applications between edge and user layers. In the edge layer, users connect with edge nodes through base stations and wireless channels. In our model, the edge layer is composed of several small areas according to the locations of edge nodes, and each of them is independent. The edge nodes are heterogeneous, in that they own different capacities. Let $V=\{V_{i}\}$ denote the set of edge nodes, where $V_{i}$ represents the $i^{th}$ one. We use $C_{i}$ to represent the computing capacity of edge node $V_{i}$. In the user layer, users are connecting with the edge node located in their area. Here, the users are local devices, such as mobile phones, laptops, smart bracelets, and so on. Let $U_{i}=\left\{u_{i}^{k}\right\}$ represent the set of users that connecting with edge node $V_{i}$. We use $u_{i}^{k}$ to denote the $k^{th}$ user in set $U_{i}$. We use $c_{i}^{k}$ to denote the computing capacity of $u_{i}^{k}$. The main notations that are commonly used throughout the paper are listed in Table 1.

#### 3.1.1. Application Model

In this paper, we assume that the applications are generated by the set of users which composed of several fine-grained sub-tasks. We use a Directed Acyclic Graph (DAG) to represent the application. Let $A_{i}^{k}=\left\{A_{i}^{k},E_{i}^{k}\right\}$ denote the application generated by $u_{i}^{k}$, where $A_{i}^{k}=\left\{a_{i}^{k}\left(1\right),a_{i}^{k}\left(2\right),...,a_{i}^{k}\left(l\right),...,a_{i}^{k}\left(n\right)\right\}$ is the set of sub-tasks. $a_{i}^{k}\left(l\right)$ denotes the $l^{th}$ sub-task. We use a vector to describe the demand of $a_{i}^{k}\left(l\right)$, where $a_{i}^{k}\left(l\right)=\langle w_{i}^{k}\left(l\right),\delta_{i}^{k}\left(l\right),t_{i}^{k}\left(l\right)\rangle$. Here, $w_{i}^{k}\left(l\right)$ refers to the workload of $v_{i}^{k}\left(l\right)$, which indicates the CPU clock cycles required to execute sub-task $a_{i}^{k}\left(l\right)$. $\delta_{i}^{k}\left(l\right)$ indicates the ratio of the output data size to the sub-task $a_{i}^{k}\left(l\right)$. $t_{i}^{k}\left(l\right)$ refers to the maximum tolerant delay. We use a boolean variable $\zeta_{i}^{k}\left(l\right)$ to record the offloading decision, where $\zeta_{i}^{k}\left(l\right)=\left\{0,1\right\}$. When the sub-task $v_{i}^{k}\left(l\right)$ execute locally, $\zeta_{i}^{k}\left(l\right)=0$; otherwise, $\zeta_{i}^{k}\left(l\right)=1$.

#### 3.1.2. Transmission Model

The transmission model is defined for the condition that sub-tasks offloading on the edge nodes. According to the Rayleigh fading channel model in Reference [24], the rate of $u_{i}^{k}$ that transmits $a_{i}^{k}\left(l\right)$ to the edge node $V_{j}$ is defined as

(1) $$r_{a_{i}^{k}\left(l\right),V_{j}}=B_{i,j}\cdot \log_{2}\left(1+\frac{p_{i,j}h_{i,j}}{d_{i,j}^{\omega_{i}}N_{i}}\right),$$

where $B_{i,j}$ represents the transmission bandwidth between $u_{i}^{k}$ and $V_{j}$, and $p_{i,j}$ represents the transmission power from $u_{i}^{k}$ to $V_{j}$. $h_{i,j}$ and $d_{i,j}^{\omega_{i}}$ represent the channel gain and distance between $u_{i}^{k}$ and $V_{j}$, respectively. $\omega_{i}$ denotes the path loss exponent, and $N_{i}$ denotes the Gaussian noise.

#### 3.1.3. Execution on Local Devices

We consider the offloading problem for the fine-grained sub-tasks that decide to perform either locally or edge nodes. We first discuss the total delay when the sub-tasks execute on local devices. For each sub-task, the total delay consists of two components, which are the computing delay and the waiting delay. In the application model, we use $w_{i}^{k}\left(l\right)$ to denote the workload of $a_{i}^{k}\left(l\right)$, which indicates the CPU clock cycles required to execute. The computing delay $D_{local}^{e}\left(a_{i}^{k}\left(l\right)\right)$ is defined as:

(2) $$D_{local}^{e}\left(a_{i}^{k}\left(l\right)\right)=\frac{w_{i}^{k}\left(l\right)}{c_{i}^{k}}.$$

There are two scenarios of waiting delays in local: one is the waiting delay for the execution of the *k* predecessor sub-tasks and returning the results, and the other one is the delay of waiting for the local execution of the sub-tasks. We use $D_{local}^{p}\left(a_{i}^{k}\left(l\right)\right)$ to denote the waiting delay for the execution of the *k* predecessor sub-tasks, and $D_{local}^{r}\left(a_{i}^{k}\left(l\right)\right)$ to denote the delay of returning the results.

(3) $$D_{local}^{r}\left(a_{i}^{k}\left(l\right)\right)=\frac{pre\left(\frac{w_{i}^{k}\left(l\right)}{\eta_{i}^{k}\left(l\right)}\right)\cdot \delta_{i}^{k}\left(l\right)}{r_{a_{i}^{k}\left(l\right),V_{j}}}.$$

$pre(\frac{w_{i}^{k}\left(l\right)}{\eta_{i}^{k}\left(l\right)})$ represents the data size of precursor sub-tasks of $a_{i}^{k}\left(l\right)$, where $\eta_{i}^{k}\left(l\right)$ denotes the CPU cycles required for each MB of the sub-task $a_{i}^{k}\left(l\right)$. $\delta_{i}^{k}\left(l\right)$ represents the ratio of the output data size to the sub-task $a_{i}^{k}\left(l\right)$. $r_{a_{i}^{k}\left(l\right),V_{j}}$ is the transmission rate that transmits $a_{i}^{k}\left(l\right)$ to the edge node $V_{j}$. We use $D_{local}^{q}\left(a_{i}^{k}\left(l\right)\right)$ to denote the queuing delay of local execution of *k* predecessor sub-tasks.

Therefore, the total waiting delay $D_{local}^{w}\left(a_{i}^{k}\left(l\right)\right)$ is defined as

(4) $$D_{local}^{w}\left(a_{i}^{k}\left(l\right)\right)=\max\left\{D_{local}^{p}\left(a_{i}^{k}\left(l\right)\right)+D_{local}^{r}\left(a_{i}^{k}\left(l\right)\right),D_{local}^{q}\left(a_{i}^{k}\left(l\right)\right)\right\}.$$

The total delay is defined as

(5) $$D_{local}\left(a_{i}^{k}\left(l\right)\right)=D_{local}^{w}\left(a_{i}^{k}\left(l\right)\right)+D_{local}^{e}\left(a_{i}^{k}\left(l\right)\right).$$

The total energy consumption for sub-task $a_{i}^{k}\left(l\right)$ on $u_{i}^{k}$ is defined as

(6) $$E_{local}\left(v_{i}^{k}\left(l\right)\right)=\epsilon_{i}^{k}\cdot w_{i}^{k}\left(l\right)\cdot {\left(c_{i}^{k}\right)}^{2}.$$

Here, $\epsilon_{i}^{k}$ is the coefficient factor [25] of chip architecture on $u_{i}^{k}$.

#### 3.1.4. Execution on Edge Nodes

Comparing with the sub-tasks executing locally, the total delay under the edge nodes includes the transmission delay. We use $D_{edge}^{t_{i,j}}\left(a_{i}^{k}\left(l\right)\right)$ to represent the transmission delay from $u_{i}^{k}$ to $V_{j}$.

(7) $$D_{edge}^{t_{i,j}}\left(a_{i}^{k}\left(l\right)\right)=\frac{w_{i}^{k}\left(l\right)}{\eta_{i}^{k}\left(l\right)\cdot r_{a_{i}^{k}\left(l\right),V_{j}}}.$$

We use $\eta_{i}^{k}\left(l\right)$ to denote the CPU cycles required for each MB of the sub-task $a_{i}^{k}\left(l\right)$. Since $w_{i}^{k}\left(l\right)$ is the workload of sub-task $a_{i}^{k}\left(l\right)$, the data size is $\frac{w_{i}^{k}\left(l\right)}{\eta_{i}^{k}\left(l\right)}$.

The computing delay $D_{edge}^{e}\left(a_{i}^{k}\left(l\right)\right)$ is defined as:

(8) $$D_{edge}^{e}\left(a_{i}^{k}\left(l\right)\right)=\frac{w_{i}^{k}\left(l\right)}{C_{j}}.$$

$C_{j}$ is the computing capacity of edge node $V_{j}$.

**Figure 2:**
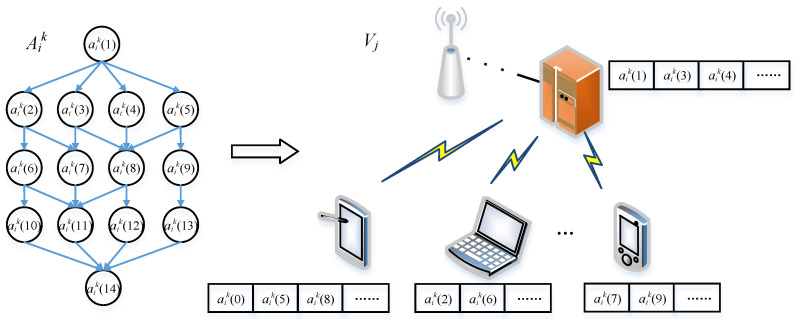
An abstraction of the system model.

In this case, the waiting delay for the sub-task $a_{i}^{k}\left(l\right)$ involves the preparation time for the precursor sub-tasks of $pre(a_{i}^{k}\left(l\right))$ and the return time of the result. In addition, we suppose that the capacities of the edge nodes are also limited, and one edge node can only execute one sub-task at the same time. The abstract model is shown in Figure 2. The waiting delay is defined as

(9) $$D_{edge}^{w}\left(a_{i}^{k}\left(l\right)\right)=\max\left\{D_{edge}^{p}\left(a_{i}^{k}\left(l\right)\right)+D_{edge}^{r}\left(a_{i}^{k}\left(l\right)\right),D_{edge}^{q}\left(a_{i}^{k}\left(l\right)\right),D_{edge}^{t_{i,j}}\left(a_{i}^{k}\left(l\right)\right)\right\}.$$

$D_{edge}^{p}\left(a_{i}^{k}\left(l\right)\right)$ and $D_{edge}^{r}\left(a_{i}^{k}\left(l\right)\right)$ are the preparation time for the precursor sub-tasks of $pre(a_{i}^{k}\left(l\right))$ and the return time of the result, respectively. The total delay is defined as

(10) $$D_{edge}\left(a_{i}^{k}\left(l\right)\right)=D_{edge}^{w}\left(a_{i}^{k}\left(l\right)\right)+D_{edge}^{e}\left(a_{i}^{k}\left(l\right)\right).$$

The total energy consumption for sub-task $a_{i}^{k}\left(l\right)$ on edge node $V_{j}$ is defined as

(11) $$E_{edge}\left(a_{i}^{k}\left(l\right)\right)=p_{i}\cdot D_{edge}^{t_{i,j}}\left(a_{i}^{k}\left(l\right)\right)+p_{j}\cdot D_{r}\left(a_{i}^{k}\left(l\right)\right).$$

Here, $p_{j}$ and $p_{j}$ are the transmission power of local device $u_{i}^{k}$ and edge node $V_{j}$, respectively.

Since the types of sub-tasks vary according to the application scenarios. Some of them are sensitive to the delay, while others are more sensitive to the energy consumption. Therefore, we jointly consider the total delay and energy consumption. Let $D_{i}^{k}$ denote the total delay of user $u_{i}^{k}$, where

(12) $$D_{i}^{k}=\zeta_{i}^{k}\left(l\right)\cdot D_{edge}\left(a_{i}^{k}\left(l\right)\right)+\left(1-\zeta_{i}^{k}\left(l\right)\right)D_{local}\left(a_{i}^{k}\left(l\right)\right).$$

Let *E* denote the total energy consumption, where

(13) $$E_{i}^{k}=\zeta_{i}^{k}\left(l\right)\cdot E_{edge}\left(a_{i}^{k}\left(l\right)\right)+\left(1-\zeta_{i}^{k}\left(l\right)\right)E_{local}\left(a_{i}^{k}\left(l\right)\right).$$

Therefore, the total cost of user $u_{i}^{k}$ is defined as

(14) $$T_{i}^{k}=\rho \cdot D_{i}^{k}+\left(1-\rho \right)E_{i}^{k}.$$

In this paper, we consider the delay and energy consumption of applications generated by users. Our objective is to minimize the total cost, and the formulation is shown as follows:

(15) $$\begin{array}{cc} \mathrm{minimize} & \sum_{i=1}^{\left|V\right|}\sum_{k=1}^{|U_{i}|}T_{i}^{k}, \end{array}$$

(16) $$\begin{array}{cc} \mathrm{subject}\;\mathrm{to} & D_{i}^{k}\leq \tau_{i}^{k},\forall u_{i}^{k}\in U_{i}, \end{array}$$

(17) $$\begin{array}{cc} & \left(a_{i}^{k}\left(l-1\right),a_{i}^{k}\left(l\right)\right)\in A_{i}^{k}, \end{array}$$

(18) $$\begin{array}{cc} & \zeta_{i}^{k}\left(l\right)=\left\{0,1\right\}. \end{array}$$

Equation (15) represents the objective function, and Equations (16)–(18) are the constrains. Equation (16) represents the total delay of an application requires that should not exceed the maximum required delay. Equation (17) represents the dependency of the sub-tasks in the application, and Equation (18) represents the constraints on the locations of the offloading, where 1 denotes that representing to offload on the edge node, otherwise to the local devices.

## 4. An Application-Driven Task Offloading Strategy Based on DRL

In this section, we propose an Application-driven Task Offloading Strategy (ATOS) based on DRL. The main idea of ATOS is to add a preliminary sorting mechanism and realize the jointly optimization of the delay and energy consumption by proposing a task offloading strategy based on the deep Q-learning. The detailed description of ATOS is shown as follows.

### 4.1. Preliminary Sorting Mechanism (PSM)

**Figure 3:**
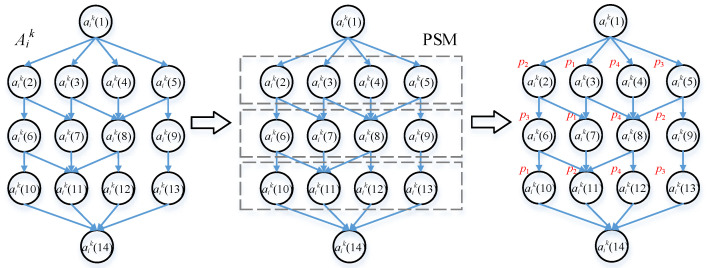
An illustration of PSM for the application.

In this paper, we assume that the applications are generated by the users which are composed of several fine-grained sub-tasks. Although these sub-tasks have strong dependencies, there are still existing some parallel sub-tasks whose execution order will affect the result of subsequent task offloading. An illustration of PSM for the application $A_{i}^{k}$ is shown in Figure 3. In this subsection, we introduce a Preliminary Sorting Mechanism (PSM) to determine the sequences of sub-tasks. We first initialize the preliminary sorting set $\omega_{i}^{k}=:\Phi$ in line 1. For each sub-task $a_{i}^{k}$ in application $A_{i}^{k}$, we check the in-degree $I(a_{i}^{k})$. If the value of in-degree is 0, we add this sub-task into queue $S_{i}^{k}$. Otherwise, we check the out-degree $O(a_{i}^{k})$. If the value of out-degree is 0, it represents that this is the last sub-task in the application. Then, we return the sequence queue $S_{i}^{k}$. If neither of the above cases is true, we add the subsequent and sibling sub-tasks of $sub(a_{i}^{k})$ to the preliminary sorting set $\omega_{i}^{k}$. According to the structure and characteristics of the application, we define a priority factor $f_{i}^{k}$.

**Definition** **1** (**Priority Factors**)**.** *The priority factors $f_{i}^{k}$ is to decide the execution order for the parallel sub-tasks in application $A_{i}^{k}$, where $f_{i}^{k}\left(l\right)=\frac{I(a_{i}^{k}\left(l\right))}{O\left(a_{i}^{k}\left(l\right)\right)\cdot w_{i}^{k}\left(l\right)}$.*

Based on that, we calculate the priority factors $f_{i}^{k}$ for subsequent sub-tasks in set $\omega_{i}^{k}$. In line 9, we update $\omega_{i}^{k}$ with descending order of $f_{i}^{k}$, where $\omega_{i}^{k}:=descending\left(sub\left(a_{i}^{k}\right)\right)$. Then, we update $S_{i}^{k}$ by adding the preliminary sorting set $\omega_{i}^{k}$ into queue. In line 11, we return sequence queue $S_{i}^{k}$.

### 4.2. Task Offloading Based on Deep Q-Learning

In this subsection, we introduce our task offloading strategy based on DQN. To describe the environment of the DCN correctly and concisely for the agent, the state space should include the knowledge of applications and the status of the total cost. So, the state is designed as follows.

**Definition** **2** (**State**)**.** *The state $s_{t}$ is a vector consisting of $s_{t}={\left[T_{i}^{k},U_{i}/\hat{U_{i}}\right]}_{t}$, where $U_{i}/\hat{|U_{i}|}$ are the sub-tasks waiting to be scheduled, and $T_{i}^{k}=\sum_{k=1}^{\hat{|U_{i}|}}T_{i}^{k}$ is the total cost of the scheduled sub-tasks $\hat{U_{i}}$.*

We consider realizing the offloading by training the agent which needs to choose a destination (edge nodes or local devices) for the sub-tasks of each application. The action $A_{t}$ is designed as follows.

**Definition** **3** (**Action**)**.** *The action space $a_{t}={\left[\zeta_{i}^{k}\left(l\right),1-\zeta_{i}^{k}\left(l\right)\right]}_{t}$ is the adjusting action, where $\zeta_{i}^{k}\left(l\right)=0$ or $\zeta_{i}^{k}\left(l\right)=1$ means that the target location of adjustment is on edge node or local device.*

At each time slot *t*, the agent will receive a reward $R(s_{t},a_{t})$ in a certain state st after executing action $a_{t}$. Since the objective is to minimize the total cost of delay and energy consumption which contract with the goal of RL that maximizing the long-term reward, the reward function should be negatively related to the weighted sum of delay and energy consumption. The reward function $R(s_{t},a_{t})$ is designed as follows.

**Definition** **4** (**Reward**)**.** *The immediate reward is $R\left(s_{t},a_{t}\right)=\frac{T_{base}-T_{i}^{k}}{T_{base}}$, where $T_{i}^{k}$ is the total cost of the scheduled sub-tasks, and $T_{base}$ is a baseline cost that offloading with greedy strategy.*

Algorithm 1 summarizes the ATOS, and the main idea is to use a deep reinforcement learning agent to perform the dynamic offloading of sub-tasks in applications to minimize the total cost of delay and energy consumption. We first initialize some preliminary parameters which include setting the replay memory D to capacity *N*. Meanwhile, we initialize the action-value function *Q* with random weight θ and the target action-value function $\hat{Q}$ with weights $\theta^{-}=\theta$. In lines 2 to 15, we start to train the agent by running a number of κ episodes with our environment. During each episode, Initialize sequence $S_{i}^{k}$ based on Algorithm 2 in line 3. The training process starts from lines 4 to 14. In line 4, the agent selects a random action $a_{t}$ with probability ε; otherwise, it will select $a_{t}=argmax_{a}Q\left(\phi \left(s_{t}\right),a;\theta \right)$ with the maximum *Q* value in line 5. In line 6, we set $s_{t+1}=s_{t}$, $a_{t}$, $x_{t+1}$, and preprocess $\phi_{t+1}=\phi \left(S_{t+1}\right)$, and we store the transition $\left(\phi_{t},a_{t},r_{t},\phi_{t+1}\right)$ in the replay memory D. After that, we sample a random minibatch of transitions $\left(\phi_{j},a_{j},r_{j},\phi_{j+1}\right)$ from D in lines 7 to 8. The objective of our problem is to minimize the total cost of the users which is contrary to the cumulative reward received by the agent. In line 12 to 14, the agent performs a gradient descent step on ${\left(y-Q\left(\phi_{j},a_{j};\theta \right)\right)}^{2}$ with respect to the network parameters θ, and resets $\hat{Q}=Q$ every *C* steps. The offloading results are returned in line 15.
**Algorithm 1** Application-driven Task Offloading Strategy based on DQN (ATOS).**Input:** The applications $A_{i}^{k}$ generated by user $U_{i}$ with sequences $S_{i}^{k}$; **Output:** Offloading strategy $X_{i}^{k}$;
1:Initialize D to *N*, *Q* with random weights θ, and $\hat{Q}$ with weights $\theta^{-}:=\theta$;2:**for** episode from 1 to κ **do**3:  Initialize sequence $S_{i}^{k}$ based on Algorithm 2;4:  With probability ε select a random action $a_{t}$;5:  Otherwise select $a_{t}=argmax_{a}Q\left(\phi \left(S_{t}\right),a;\theta \right)$;6:  Set $S_{t+1}=S_{t}$, $a_{t}$, $x_{t+1}$ and preprocess $\phi_{t+1}=\phi \left(S_{t+1}\right)$.7:  Store transition $\left(\phi_{t},a_{t},r_{t},\phi_{t+1}\right)$ in D;8:  Sample random minibatch of transitions $\left(\phi_{j},a_{j},r_{j},\phi_{j+1}\right)$ from D.9:  **if** episode terminates at step j+1 **then**10:    Set $y_{j}=r_{j}$;11:  **else**12:    Set $y_{j}=r_{j}+\gamma max_{a^{\prime }}\hat{Q}\left(\phi_{j+1},a^{\prime };\theta^{-}\right)$;13:    Perform a gradient descent step on ${\left(y-Q\left(\phi_{j},a_{j};\theta \right)\right)}^{2}$ with respect to the network parameters θ.14:    Every *C* steps reset $\hat{Q}=Q$;15:**return** Offloading strategy $X_{i}^{k}$;


**Algorithm 2** Preliminary Sorting Mechanism (PSM).**Input:** The application $A_{i}^{k}$ generated by user $U_{i}$; **Output:** The sequence queue $S_{i}^{k}$ of the sub-tasks in $A_{i}^{k}$;
1:Initialize the preliminary sorting set $\omega_{i}^{k}=:\Phi$;2:**for** each sub-task $a_{i}^{k}$ in $A_{i}^{k}$ **do**3:  **if** $I(a_{i}^{k})=0$ **then**4:    Adding sub-task $a_{i}^{k}$ into queue $S_{i}^{k}$;5:  **else if** $O(a_{i}^{k})=0$ **then**6:    Go to line 11;7:  Adding subsequent and sibling sub-tasks of $sub(a_{i}^{k})$ to preliminary sorting set $\omega_{i}^{k}$;8:  Calculate the priority factors $f_{i}^{k}$ for subsequent sub-tasks in set $\omega_{i}^{k}$;9:  Update $\omega_{i}^{k}$ with descending order of $f_{i}^{k}$, where $\omega_{i}^{k}:=descending\left(sub\left(a_{i}^{k}\right)\right)$;10:   Update $S_{i}^{k}$ by adding set $\omega_{i}^{k}$ into queue;11:**return** Sequence queue $S_{i}^{k}$;


## 5. Experiment Evaluation

In this section, we will conduct experiments on the designing simulator to evaluate our strategy ATOS. We analyzed and shown the experimental results from different perspectives to provide insightful conclusions.

### 5.1. Basic Setting of the Synthetic Dataset

In this subsection, we develop a simulator using python and evaluate the performance of our algorithms by building a synthetic dataset. In our simulator, the number of edge nodes ranges from 5 to 10. For each edge node, we consider an area with 500 square meters, and there are existing 1 to 5 users. The setting of parameters in our paper are listed in Table 2, which refer to References [24,26]. Each user deploys one application, and each application consists of 12 to 21 sub-tasks. In our experiments, we test several groups of hyperparameters that the learning rates range from 0.0005 to 0.001, and the e-greedy factors range from 0.7 to 0.95. In addition, we test the reward decay between 0.6 and 0.9 at 0.05 intervals, and we test the replacing target iterations between 20 and 500 at 10 intervals. According to the test results, we choose the group of hyperparameters listed in Table 3 as the experimental setting. We consider the four baseline algorithms to be the comparisons as follows: (i) Offloading all sub-tasks on the edge nodes (Offloading_edge): for each application, we offload the sub-task on the edge nodes iteratively. (ii) Offloading all sub-tasks on the local devices (Offloading_local): for each application, we offload the sub-task on the local devices iteratively. (iii) Offloading all sub-tasks on the edge nodes or the local devices randomly (Offloading_random): for each application, we offload the sub-task to the edge node or the local device randomly in each iteration. (iv) Offloading all sub-tasks on the edge nodes or the local devices through greedy strategy (Offloading_greedy): for each application, we greedy choose the offloading destination by considering the queueing time and the capacities in each iteration. We compare ATOS with these four baseline algorithms, and the effectiveness of ATOS is verified.

### 5.2. Evaluations on the Performance

**Figure 4:**
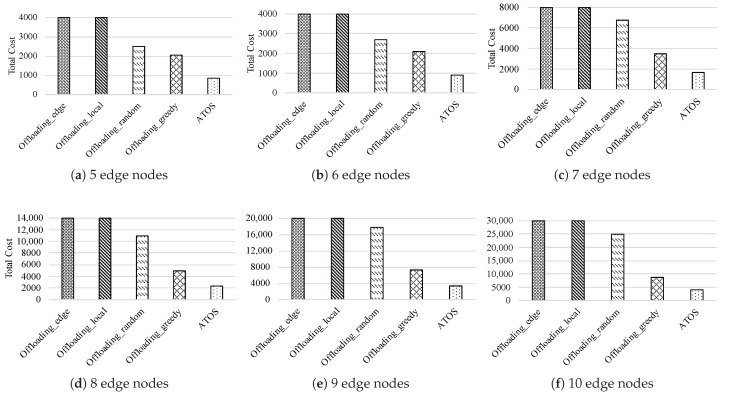
The total cost under different offloading strategies with edge nodes ranging from 5 to 10.

In this subsection, we discuss the total cost of multiple users with the application-driven task offloading requests in edge computing, the results are shown in Figure 4. Four baseline algorithms (Offloading_edge, Offloading_local, Offloading_random, Offloading_greedy) are used to compare with our algorithm. We choose 6 groups of topologies that the edge nodes in the edge layers are ranging from 5 to 10. In order to facilitate the analysis of the results, each group ran 10 times and calculated the average value. According to the results, we obtain the following observations: (i) For each group, the total cost is the largest when all tasks are executed locally (Offloading_local) or the edge nodes (Offloading_edge). As shown in Figure 4a–f, the total cost of each group for the users in both cases will reach the highest value of the ordinate. Here, in order to show the difference between the results of these two strategies and those of other ones, we set the highest limitation of the ordinate. The total costs under these six groups are listed in Table 4. We can see that, since the limited capacities of edge nodes and local devices, the total costs of these two strategies are much higher than that of other ones. In addition, the total cost of Offloading_edge is lower than Offloading_local. The reason is that, although they will produce transmission energy consumption for the sub-tasks that are offloading to the edge nodes, the high computation delay caused by limited computing capacities of local devices is the key factor of the high total cost for the users. (ii) The impact of algorithms on the total costs is related to the number of edge nodes. We compared the last three columns of the six experiments in Figure 4a–f, the trend of the total costs decreases. For the topology with a small number of edge nodes (5 and 6 edge nodes in Figure 4a,b), the gap in the total cost between Offloading_random and Offloading_greedy is not large. However, with the scaling of the topology, the total cost of Offloading_greedy is significantly lower than Offloading_random. We can see that ATOS can better reduce the total cost in the six groups. Compared with Offloading_random and Offloading_greedy, the optimization rates of ATOS are improving 78.3% and 50.6% on average, respectively.

### 5.3. Evaluations on the Convergence

**Figure 5:**
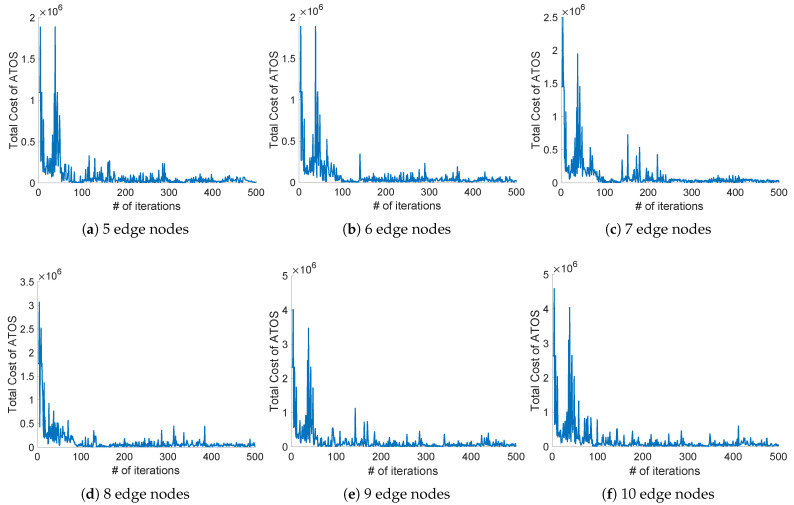
The convergence of total cost under ATOS with the number of edge nodes ranging from 5 to 10.

In this subsection, we analyze the convergence of ATOS. We choose 6 groups of topologies with different edge nodes (5, 6, 7, and 8), and the number of sub-tasks in each application ranges from 12 to 21. In order to facilitate the analysis of the results, the number of iterations of each group is 500. According to the results, we obtain the following observations: (i) The total cost under each group will close to convergence after 500 iterations. As shown in Figure 5, the total cost within 0 to 100 iterations is decreasing sharply. In groups 5, 6, 7, 9, and 10, the total cost fluctuates strongly at about 30 iterations. In group 8, although the fluctuation is not violent, the abnormal values appears frequently, at about 100 to 380 iterations. For different topologies, the ranges of convergence values are different due to the various sizes of sub-tasks in the applications. (ii) The learning ranges of ATOS increase with the expansion of different topologies. With the increasing number of edge nodes, more actions can be selected in the process of learning and training, so the range of total cost becomes larger. When the number of nodes in the edge layer reaches 10, the total cost will be close to $5\times 10^{6}$. Due to the differences in the applications deployed by users, some groups will fluctuate during the convergence process. For example, in Figure 5c, the value is fluctuating from 100 to 200 iterations. In summary, we can see that ATOS basically reaches convergence and maintains stability quickly.

## 6. Conclusions

In this paper, we study the application-driven task offloading in edge computing by considering the strong dependencies of sub-tasks. We first formulate the task offloading as a joint optimization problem that considers the total delay and energy consumption. Based on that, we propose a novel task offloading strategy ATOS based on DRL by adding a preliminary sorting mechanism. We analyze the characteristics of application-driven tasks and propose a heuristic algorithm PSM to determine the processing order of the parallelism sub-tasks. Finally, we study the convergence and performance of ATOS through extensive experiments. The results show that ATOS can obtain a reasonable offloading strategy and reduce the total cost of users.

In future work, we will consider the mobility of users under the cooperation of edge nodes. In addition, we will further consider the application-driven tasks with strong dependencies that combine with actual scenarios. 

## Figures and Tables

**Table 1:** Notations.

Symbols	Definitions
V	Set of edge nodes, where $V=\{V_{i}\}$.
$V_{i}$	The $i^{th}$ edge node set V.
$C_{i}$	The computing capacity of $V_{i}$.
$U_{i}$	Set of users that connecting with edge node $V_{i}$, where $U_{i}=\left\{u_{i}^{k}\right\}$.
$u_{i}^{k}$	The $k^{th}$ user in set $U_{i}$.
$c_{i}^{k}$	The computing capacity of $u_{i}^{k}$.
$A_{i}^{k}$	The application generated by $u_{i}^{k}$, where $A_{i}^{k}=\left\{A_{i}^{k},E_{i}^{k}\right\}$.
$A_{i}^{k}$	The set of sub-tasks in $A_{i}^{k}$, where $A_{i}^{k}=\left\{a_{i}^{k}\left(1\right),a_{i}^{k}\left(2\right),\ldots ,a_{i}^{k}\left(l\right),\ldots ,a_{i}^{k}\left(n\right)\right\}$.
$r_{a_{i}^{k}\left(l\right),V_{j}}$	The rate of $u_{i}^{k}$ that transmits $a_{i}^{k}\left(l\right)$ to $V_{j}$.
$D_{i}^{k}$	The total delay of user $u_{i}^{k}$.
$E_{i}^{k}$	The total energy consumption of user $u_{i}^{k}$.

**Table 2:** Setting of parameters.

Parameters	Values
Transmission bandwidth $B_{i,j}$	180 kHz
Path loss exponent $\omega_{i}$	3
Gaussian noise $N_{i}$	$10^{-13}$
Data size of sub-tasks	0.3 Mb∼1 Mb.
Transmission power of local device $p_{i,j}$	3 W
Computing capacity of local devices	0.5–1 GHz
Computing capacity of edge nodes	5 GHz
The coefficient of channel fading	$10^{-6}$
The coefficient factor of chip architecture	$10^{-20}$

**Table 3:** Hyperparameter settings.

Hyperparameter	Settings
learning rate α	0.0005
e-greedy ϵ	0.9
reward decay γ	0.7
replacing target iterations	30
replay memory D	500

**Table 4:** Total costs of Offloading_edge and Offloading_local.

# of Edge Nodes	Offloading_Edge	Offloading_Local
5	1,094,706	5,472,686
6	1,095,779	5,478,050
7	1,451,771.14	7,257,740.15
8	1,766,995.48	8,833,583.48
9	2,322,826.54	11,612,323.16
10	2,638,105.86	13,188,441.59
